# Insulin resistance in adolescents with Down syndrome: a cross-sectional study

**DOI:** 10.1186/1472-6823-5-6

**Published:** 2005-06-17

**Authors:** Cristina T Fonseca, Daniela M Amaral, Márcia G Ribeiro, Izabel CR Beserra, Marília M Guimarães

**Affiliations:** 1 Post-Graduate Program of Endocrinology, Medicine School, Hospital Universitário Clementino Fraga Filho (HUCFF), Federal University of Rio de Janeiro (UFRJ) - Av. Brigadeiro Trompowski, s/n, HUCFF, Ilha do Fundão, Rio de Janeiro, Brazil; 2 Genetics Department, Instituto de Puericultura e Pediatria Martagão Gesteira (IPPMG), UFRJ - Av. Brigadeiro Trompowski, s/n, HUCFF, Ilha do Fundão, Rio de Janeiro, Brazil; 3 Pediatrics Department, IPPMG, UFRJ - Av. Brigadeiro Trompowski, s/n, HUCFF, Ilha do Fundão, Rio de Janeiro, Brazil; 4 Endocrinology Department - HUCFF, UFRJ - Av. Brigadeiro Trompowski, s/n, HUCFF, Ilha do Fundão, Rio de Janeiro, Brazil

## Abstract

**Background:**

The prevalence of diabetes mellitus is higher in individuals with Down syndrome (DS) than in the general population; it may be due to the high prevalence of obesity presented by many of them. The aim of this study was to evaluate the insulin resistance (IR) using the HOMA (*Homeostasis Model Assessment*) method, in DS adolescents, describing it according to the sex, body mass index (BMI) and pubertal development.

**Methods:**

15 adolescents with DS (8 males and 7 females) were studied, aged 10 to 18 years, without history of disease or use of medication that could change the suggested laboratory evaluation. On physical examination, the pubertal signs, *acanthosis nigricans (AN)*, weight and height were evaluated. Fasting plasma glucose and insulin were analysed by the colorimetric method and RIA-kit LINCO, respectively. IR was calculated using the HOMA method. The patients were grouped into obese, overweight and normal, according to their BMI percentiles. The EPIINFO 2004 software was used to calculate the BMI, its percentile and Z score.

**Results:**

Five patients were adults (Tanner V or presence of menarche), 9 pubertal (Tanner II – IV) and 1 prepubertal (Tanner I). No one had *AN*. Two were obese, 4 overweight and 9 normal. Considering the total number of patients, HOMA was 1.7 ± 1.0, insulin 9.3 ± 4.8 μU/ml and glucose 74.4 ± 14.8 mg/dl. The HOMA values were 2.0 ± 1.0 in females and 1.5 ± 1.0 in males. Considering the nutritional classification, the values of HOMA and insulin were: HOMA: 3.3 ± 0.6, 2.0 ± 1.1 and 1.3 ± 0.6, and insulin: 18.15 ± 1.6 μU/ml, 10.3 ± 3.5 μU/ml and 6.8 ± 2.8 μU/ml, in the obese, overweight and normal groups respectively. Considering puberty, the values of HOMA and insulin were: HOMA: 2.5 ± 1.3, 1.4 ± 0.6 and 0.8 ± 0.0, and insulin: 13.0 ± 5.8 μU/ml, 7.8 ± 2.9 μU/ml and 4.0 ± 0.0 μU/ml, in the adult, pubertal and prepubertal groups respectively.

**Conclusion:**

The obese and overweight, female and adult patients showed the highest values of HOMA and insulin.

## Background

Down syndrome (DS) is a common chromosomal disorder, affecting 1 per 700 live births [1]. Life expectancy has increased in the last decades, due to the improvement of the scientific knowledge about the syndrome and its acquired complications, and also, due to the easier access and the more recent diagnostic and therapeutic means for the patients, their families and the multiprofessional health team that accompany them [2,3].

Early hypotonicity and typical dimorphisms are among the clinical characteristics of the syndrome. There is a higher incidence of diseases in various organic systems, including the endocrinologic system, such as diabetes mellitus among others [4].

Diabetes mellitus has a higher prevalence in DS than in the general population [4,5]. It can appear as an autoimmune disorder (type 1 diabetes mellitus) or as a disorder in which the insulin resistance is the predominant physiopathological factor (type 2 diabetes mellitus) [6-8].

Insulin resistance may present without any clinical manifestation, often, many years before the appearance of frank diabetes mellitus, which would occur when the pancreas failed to secrete enough insulin to compensate such resistance and keep the person euglycemic [6]. Therefore, it would be interesting to know the level of insulin resistance in this syndrome, since adolescence, as diabetes mellitus evolve with several complications which would further increase the morbidity and mortality of this population. This way, preventive measures could be applied, such as giving the patient adequate dietetic orientations and leading him to an early consultation with a nutrition service, stimulating and developing special programs of physical activity, treating those who have insulin resistance with medications, which may be a therapeutic option in the future.

The aim of this study was to estimate the insulin resistance (IR) through the HOMA (*Homeostasis Model Assessment*) method, in adolescents with DS, and to describe it according to the sex, body mass index (BMI) and presence of puberty.

## Methods

A descriptive cross-sectional study was designed with 15 out patient adolescents (8 males and 7 females) with Down syndrome, aged 10 to 18 years.

Adolescents with previously known diabetes mellitus or impaired glucose tolerance (IGT) or impaired fasting glucose (IFG) were excluded from this study. Furthermore, the studied patients did not have any comorbidity and did not use any medication that could compromise the suggested laboratory evaluation, such as anti-hyperlipidemia drugs, topic or systemic glucocorticoids, anti-hypertensive drugs, sexual steroids and GnRH analogues.

The local Institutional Ethical Committee approved the study and the informed consent was obtained from all patients' parents or tutors.

The adolescents were measured and weighed; the stature was annotated in meters and the weight in kilograms. They were also submitted to physical examination, including the evaluation of the presence of *acanthosis nigricans *and the pubertal developmental degree according to Tanner's stage [9]. Considering puberty, they were grouped, as follows:

• Prepubertal – absence of sexual development;

• Pubertal – pubertal development according to Tanner's stage II to IV;

• Adult – pubertal development according to Tanner's stage V or menarche in girls.

Blood samples were collected after a 12-hour overnight fasting period, for the analysis of plasma glucose and insulin. The glucose levels were analysed by the colorimetric method, considering the normal range from 70 to 100 mg/dl, according to the American Diabetes Association, 2003. Insulin levels were analysed by a radioimmunoassay kit (Linco Research Incorporation). This kit is specific for human insulin and does not cross react with pro-insulin (<0.2%). The normal fasting range, for adults, after an 18-hour fasting period, varies from 5 to 15 μU/ml. The method's sensitivity is <2 μU/ml, specificity is 100% and the range value within and between assay variations are 3.1% and 6.0% respectively.

Insulin resistance was calculated according to the HOMA method, through the formula: {[glucose(mg/dl)/18] X insulin(μU/mL)}/ 22.5 [10].

The EPIINFO software version 2004, provided the BMI and its percentile and Z score.

The adolescents were grouped by the nutritional status according to the National Center for Health Statistics 2000 – Center for Disease Control and Prevention(CDC – NCHS) as follows [11]:

• Undernourished – BMI percentile below 5;

• Normal -BMI percentiles 5 to 85;

• Overweight -BMI percentiles 85 to 95;

• Obese – BMI percentile above 95.

## Results

The mean age of the patients was 12.9 ± 2.6 years (varying from 10 to 18 years); none had *acanthosis nigricans. *Considering puberty, 5 were adults,9 pubertal and 1 prepubertal, and regarding the nutritional status, 2 (13.3%) were obese, 4 (26.7%) overweight and 9 (60%) normal (Table 1). The mean BMI was 20.9 ± 4.3 (from 15.5 to 29.6) and the mean BMI Z score was +0.55 ± 0.9.

**Table 1:** Distribution of patients' data.

CA	Sex	BMI	BMIp	BMI Z	Puberty	Glucose (mg/dl)	Insulin (μU/ml)	HOMA
10y	F	15.7	29.01	-0.55	Pubertal	52	4.4	0.6
10y5m	M	17.8	64.79	0.38	Pubertal	71	5.4	0.9
10y7m	F	20.7	86.11	1.09	Pubertal	74	10.9	2.0
10y9m	F	19.9	80.09	0.84	Pubertal	74	13.7	2.5
10y9m	M	15.5	20.5	-0.82	Prepubertal	85	4.0	0.8
11y4m	F	26.1	96.81	1.85	Adult	60	19.3	2.9
11y11m	M	17.2	39.23	-0.27	Pubertal	72	6.0	1.1
12y4m	M	19.4	69.16	0.5	Pubertal	98	7.6	1.8
12y4m	F	16	15.17	-1.03	Pubertal	81	6.9	1.4
13y1m	M	22.6	88.02	1.18	Pubertal	53	7.4	1.0
13y1m	M	23.7	91.93	1.4	Pubertal	69	8.0	1.4
15y6m	F	19.8	44.94	-0.13	Adult	82	6.3	1.3
16y4m	F	26.3	89.93	1.28	Adult	97	15	3.6
16y10m	M	23.7	78	0.77	Adult	57	7.4	1.0
18y	M	29.6	95.93	1.74	Adult	91	17	3.8

CA = chronological age; M = male; F = female; BMIp = BMI percentile; BMI Z = BMI Z score.

Considering the total number of patients, the mean values were: HOMA = 1.7 ± 1.0 (from 0.6 to 3.8), insulin = 9.3 ± 4.8 μU/ml (from 4.0 to 19.3 μU/ml) and glucose = 74.4 ± 14.8 mg/dl (from 52 to 98 mg/dl). Considering sex, the mean values of HOMA were 2.0 ± 1.0 in females and 1.5 ± 1.0 in males.

**Figure 1:**
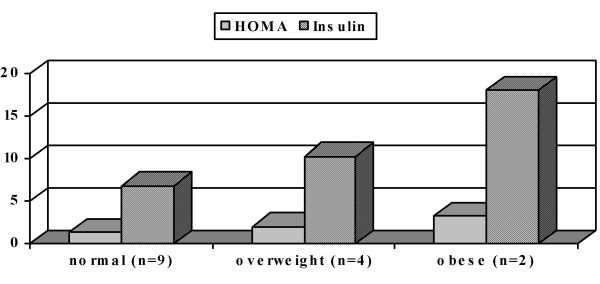
Mean values of HOMA and insulin according to the nutritional classification. The highest values of HOMA and insulin were found in the obese, followed by the overweight, and lastly, by the normal-weighed patients.

Considering the subgroups, divided by the nutritional status, the mean values were: HOMA: 3.3 ± 0.6, 2.0 ± 1.1 and 1.3 ± 0.6, and insulin: 18.15 ± 1.6 μU/ml, 10.3 ± 3.5 μU/ml and 6.8 ± 2.8 μU/ml, in the obese, overweight and normal, respectively (Figure 1).

**Figure 2:**
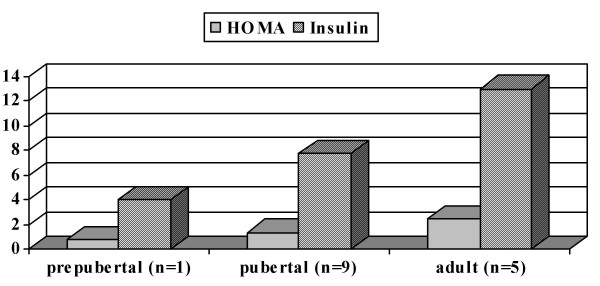
Mean values of HOMA and insulin according to the pubertal classification. The highest values of HOMA and insulin were found in the adult, followed by the pubertal, and lastly, by the prepubertal patients.

Considering puberty, the mean values were: HOMA: 2.5 ± 1.3, 1.4 ± 0.6 and 0.8 ± 0.0, and insulin: 13.0 ± 5.8 μU/ml, 7.8 ± 2.9 μU/ml and 4.0 ± 0.0 μU/ml, in the adult, pubertal and prepubertal groups, respectively (Figure 2).

## Discussion

Insulin resistance may be defined as a diminished response to the biological actions of insulin; it may involve not only the carbohydrate metabolism, but also the lipid metabolism [12]. In pharmacological terms, it represents a state in which normal amounts of insulin produce a subnormal biological response. It is characterized by hyperinsulinemia and can be associated with normoglycemia or hyperglycemia [13].

The hyperinsulinemic euglycemic clamp has been regarded as the standard method to evaluate insulin resistance. Considering that it is a difficult and relatively invasive technique that requires a specialized team and, in general, it is not available to the clinical practice [14], researchers have been studying other more practical methods that would be able to measure insulin resistance. One of these studied methods is the HOMA method, a mathematic model based on measurement of fasting plasma glucose and insulin levels, which is especially useful for DS patients in whom the use of other methods based on the results of an oral glucose tolerance test (OGTT), for example, would be extremely difficult to perform. Although it is not as sensitive or reproducible a measure of IR as the clamp technique, it has been validated both in adults [10,14] and in children and adolescents without DS [15,16] and, for this reason, it has been extensively used, not only in epidemiological studies, but also in clinical practice [10].

In Down syndrome there is a high prevalence of overweight and obesity [17-19] and one study suggests that there may be a different distribution of body fat, more truncal than peripheral, in such children, which may represent only one more trait of the syndrome or be the result of the muscle hypotonicity that is well known to be part of the syndrome's characteristics, and this may reduce the activity level and energy needs of these patients [17]. Therefore, the higher frequency of type 2 diabetes mellitus in DS may be associated not only with the higher propensity for obesity, but also with large abdominal fat stores, reflecting larger amounts of visceral adiposity, which may contribute to insulin resistance [20,21] and consequently, to the development of type 2 diabetes mellitus in a variable period of time. Such correlation between type 2 diabetes mellitus and the higher proportion of body fat mass, mainly concentrated in the abdominal region, has also been demonstrated by some studies involving type 2 diabetic, non-obese [21] or obese individuals, without DS [13].

Android obesity, that is, the obesity in which there is accumulation of fat in the central region of the body, especially in the abdomen, is more associated with insulin resistance than the gynecoid obesity [20]. It is also known that the visceral adipocytes, when compared to the subcutaneous, have a higher basal lipolysis, are poorly sensitive to insulin and that such characteristics are amplified when they show hypertrophy [22]. Consequently, this increase in lipolysis offers more free fatty acids to the liver, which stimulates, this way, the liver production of glucose and the inhibition of glucose uptake and its oxidation in muscle tissue [23]. In an attempt to keep euglycemia, insulin secretion increases and the resultant compensatory hyperinsulinemia, in obese patients, reduces the expression of the membrane insulin receptor (down regulation), which generates more resistance to the insulin action [24].

In the present study, the highest values of HOMA were found in the obese and overweight patients, but we can not affirm if such patients really have insulin resistance, as there is no consensus for ideal HOMA values for children and adolescents and much less for patients with DS. No references about insulin resistance indexes in DS were found in literature. Nevertheless, comparing our results to those showed in some studies involving youth without DS, such as the study of Yeckel *et al *[25], we could say that none of our patients had IR because such authors found mean values of HOMA of about 7.00 for normal glucose tolerant children and adolescents without DS, based on clamp and OGTT studies, while our highest HOMA value was 3.8. Nevertheless, such study involved exclusively moderate to severe obese patients whose BMI was much higher than that of our obese patients (38.1 × 27.8 respectively), fact that may have contributed to the highest values of HOMA presented by them, confirming the direct relation between obesity and HOMA values, which is in accordance with our results. Moreover, our sample involved just 2 obese patients; most of them had normal weight. On the other hand, Allard *et al *showed lower mean values of HOMA, ranging from 0.83 to 1.62 in a representative sample of 2244 children and adolescents without DS [26]. A study performed in Rio de Janeiro, Brazil, with normal-weighed healthy students, also without DS, suggested a mean value of HOMA of 2.36 for girls and 2.66 for boys, using the same method of insulin dosage as the one used in our study [27]. Based on such reference values of Brazilian children and adolescents, we found 4 (26.7%) patients (3 females and 1 male) in our study with insulin resistance when assessed through the HOMA method. Among them, 2 had obesity, 1 overweight and the other, normal weight, that is, 100% (2/2) of the obese, 25% (1/4) of the overweight and 11.1% (1/9) of the normal weight groups had insulin resistance. Unfortunately, our small sample did not permit us to confirm these data with statistical analysis as referred in previous reports that correlated obesity and insulin resistance, in obese patients, without DS [28,29].

We also found higher HOMA values in adolescents with complete pubertal development than in the pubertal ones, which was similar to literature data; this fact demonstrates that puberty represents a period marked by higher levels of insulin resistance not only in healthy adolescents, but also in those with DS [13,30]. Nevertheless, healthy adolescents have physiological compensatory mechanisms that avoid the appearance of deleterious effects in the future that could result from this reduction in insulin action, whereas in DS, it may be that mechanisms fail in the adolescent years, resulting in higher insulin resistance levels than in the healthy population of the same age. The detection of higher HOMA values in females than in males has been previously described in literature, but only in adults and in clinically healthy patients [13].

## Conclusion

We conclude that the obese and overweight, female and adult patients showed the highest values of HOMA and insulin. Nevertheless, additional studies are necessary, including larger samples and longitudinal follow-up in order to confirm the high prevalence of insulin resistance in Down syndrome, especially in the obese patients, and to verify if the patients with the highest values of HOMA may develop any glucose metabolism disturbance in the future.

## Competing interests

The author(s) declare that they have no competing interests.

## Authors' contributions

CTF and DMA selected and examined the patients, collected the blood samples, evaluated the analysis results and drafted the manuscript. MGR helped to select the patients and ICRB participated in the design of the study. MMG conceived the study and participated in its design and coordination and helped to draft the manuscript. All authors read and approved the final manuscript.

## Pre-publication history

The pre-publication history for this paper can be accessed here:



## References

[B1] Sugayama SMM, Kim CA, Setian N (2002). Anormalidades Cromossômicas. Endocrinologia Pediátrica – Aspectos físicos e metabólicos do recém-nascido ao adolescente.

[B2] Cooley WC, Graham JM (1991). Down syndrome – an update and review for the primary pediatrician. Clin Pediatr.

[B3] Baird PA, Sadovnick AD (1987). Life expectancy in Down syndrome. J Pediatr.

[B4] Smith DS (2001). Health care management of adults with Down syndrome. Am Fam Physician.

[B5] Milunsky A, Neurath PW (1968). Diabetes mellitus in Down's syndrome. Arch Environ Health.

[B6] Amiel SA, Buchanan CR, Brook CGD, Hindmarsh PC (2001). Diabetes mellitus. Clinical Pediatric Endocrinology.

[B7] Sperling MA, Sperling MA (2002). Diabetes mellitus. Pediatric Endocrinology.

[B8] Chen H Down syndrome. http://www.emedicine.com.

[B9] Marshall WA, Tanner JM (1969). Variations in the pattern of pubertal changes in girls. Arch Dis Child.

[B10] Matthews DR, Hosker JP, Rudenski AS, Naylor BA, Treacher DF, Turner RC (1985). Homeostasis model assessment: insulin resistance and β-cell function from fasting plasma glucose and insulin concentrations in man. Diabetologia.

[B11] National Center for Health Statistics. National Center for Chronic Disease Prevention and Health Promotion and Center for Disease Control and Prevention. http://www.cdc.gov/nchs/.

[B12] Wallace TM, Matthews DR (2002). The assessment of insulin resistance in man. Diabetic Medicine.

[B13] Krentz AJ (1996). Fortnightly review: insulin resistance. BMJ.

[B14] Bonora E, Targher G, Alberiche M, Bonadonna RC, Saggiani F, Zenere MB, Monauni T, Muggeo M (2000). Homeostasis model assessment closely mirrors the glucose clamp technique in the assessment of insulin sensitivity. Diabetes Care.

[B15] Gungor N, Saad R, Janosky J, Arslanian S (2004). Validation of surrogate estimates of insulin sensitivity and insulin secretion in children and adolescents. J Pediatr.

[B16] Uwaifo GI, Fallon EM, Chin J, Elberg J, Parikh SJ, Yanovski JA (2002). Indices of insulin action, disposal, and secretion derived from fasting samples and clamps in normal glucose-tolerant black and white children. Diabetes Care.

[B17] Baer MT, Waldron J, Gumm H, van Dyke DC, Chang H, van Dyke DC, Lang DJ, Heide F, van Duyne S, Soucek MJ (1990). Nutrition assessment of the child with Down syndrome. Clinical perspectives in the management of Down syndrome.

[B18] Cronk CE, Chumlea WC, Roche AF (1985). Assessment of overweight children with trisomy 21. Am J Ment Defic.

[B19] Al Husain M (2003). Body mass index for Saudi children with Down's syndrome. Acta Paediatr.

[B20] Bjorntorp P (1991). Metabolic implications of body fat distribution. Diabetes Care.

[B21] Banerji MA, Chaiken RL, Gordon D, Kral JG, Lebovitz HE (1995). Does intra-abdominal adipose tissue in black men determine whether NIDDM is insulin-resistant or insulin-sensitive?. Diabetes.

[B22] McCarty MF (2001). Modulation of adipocyte lipoprotein lipase expression as a strategy for preventing or treating visceral obesity. Med Hypotheses.

[B23] Boden G, Chen X, Ruiz J, White JV, Rossetti L (1994). Mechanisms of fatty-acid induced inhibition of glucose uptake. J Clin Invest.

[B24] Seely BL, Olefsky JM, Moller D (1993). Potential celular and genetic mechanisms for insulin resistance in the common disorders of diabetes and obesity. Insulin resistance.

[B25] Yeckel CW, Weiss R, Dziura J, Taksali SE, Dufour S, Burgert TS, Tamborlane WV, Caprio S (2004). Validation of insulin sensitivity indices from oral glucose tolerance test parameters in obese children and adolescents. J Clin Endocrinol Metab.

[B26] Allard P, Delvin EE, Paradis G, Hanley JA, O'Loughlin J, Lavallée C, Levy E, Lambert M (2003). Distribution of fasting plasma insulin, free fatty acids, and glucose concentrations and of Homeostasis Model Assessment of insulin resistance in a representative sample of Quebec children and adolescents. Clin Chem.

[B27] Pessoa CHCN, Pessoa MBHCN, Amaral DM, Teixeira AM, Cunha EF, Guimarães MM, Cordeiro JGH (2003). Estudo do Homeostasis Model Assessment (HOMA) em grupo de escolares saudáveis. Arq Bras Endocrinol Metab.

[B28] Guzzaloni G, Grugni G, Mazzilli G, Moro D, Morabito F (2002). Comparison Between β-cell Function and Insulin Resistance Indexes in Prepubertal and Pubertal Obese Children. Metabolism.

[B29] Barja S, Arteaga A, Acosta AM, Hodgson MI (2003). Resistencia Insulínica y otras expresiones del síndrome metabólico em niños obesos chilenos. Rev Méd Chile.

[B30] Amiel SA, Sherwin RS, Simonson DC, Luaritano AA, Tamborlane WV (1986). Impaired insulin action in puberty: a contributing factor to poor glycemic control during adolescence. N Engl J Med.

